# Supplementary material for: Seminoma and Embryonal Carcinoma Footprints Identified by Analysis of Integrated Genome-Wide Epigenetic and Expression Profiles of Germ Cell Cancer Cell Lines
Source: PLoS One. 2014 Jun 2;9(6):e98330. doi: 10.1371/journal.pone.0098330 (PMC4041891; doi:10.1371/journal.pone.0098330)
Supplement: File S1 — ZIP file containing DMRforPairs output for significant regions. Please start from the html files. (ZIP) [file pone.0098330.s008.zip › figures/36808.pdf]

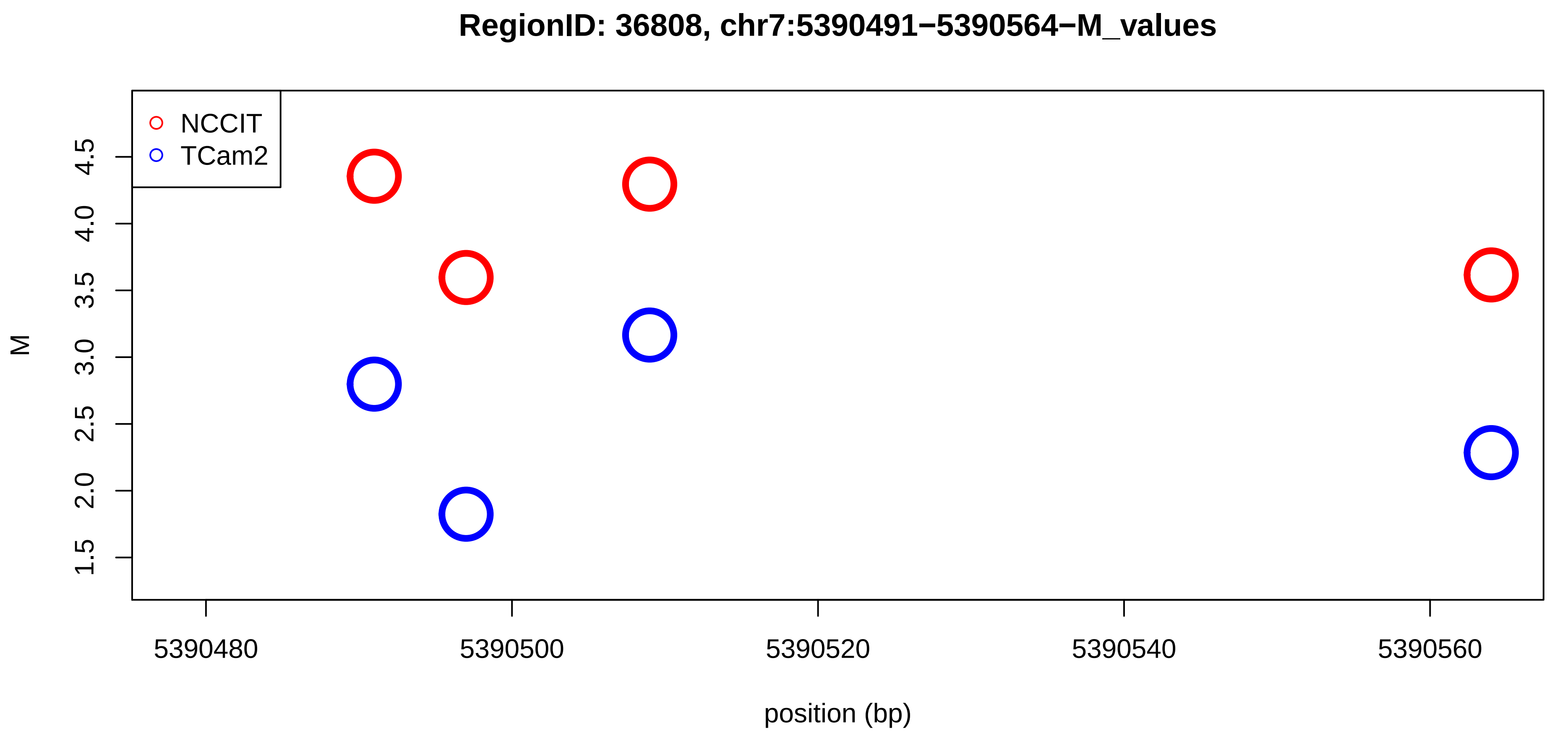
RegionID: 36808, chr7:5390491−5390564−M_values
NCCIT
TCam2
M
1.5
2.0
2.5
3.0
3.5
4.0
4.5
5390480
5390500
5390520
5390540
5390560
position (bp)

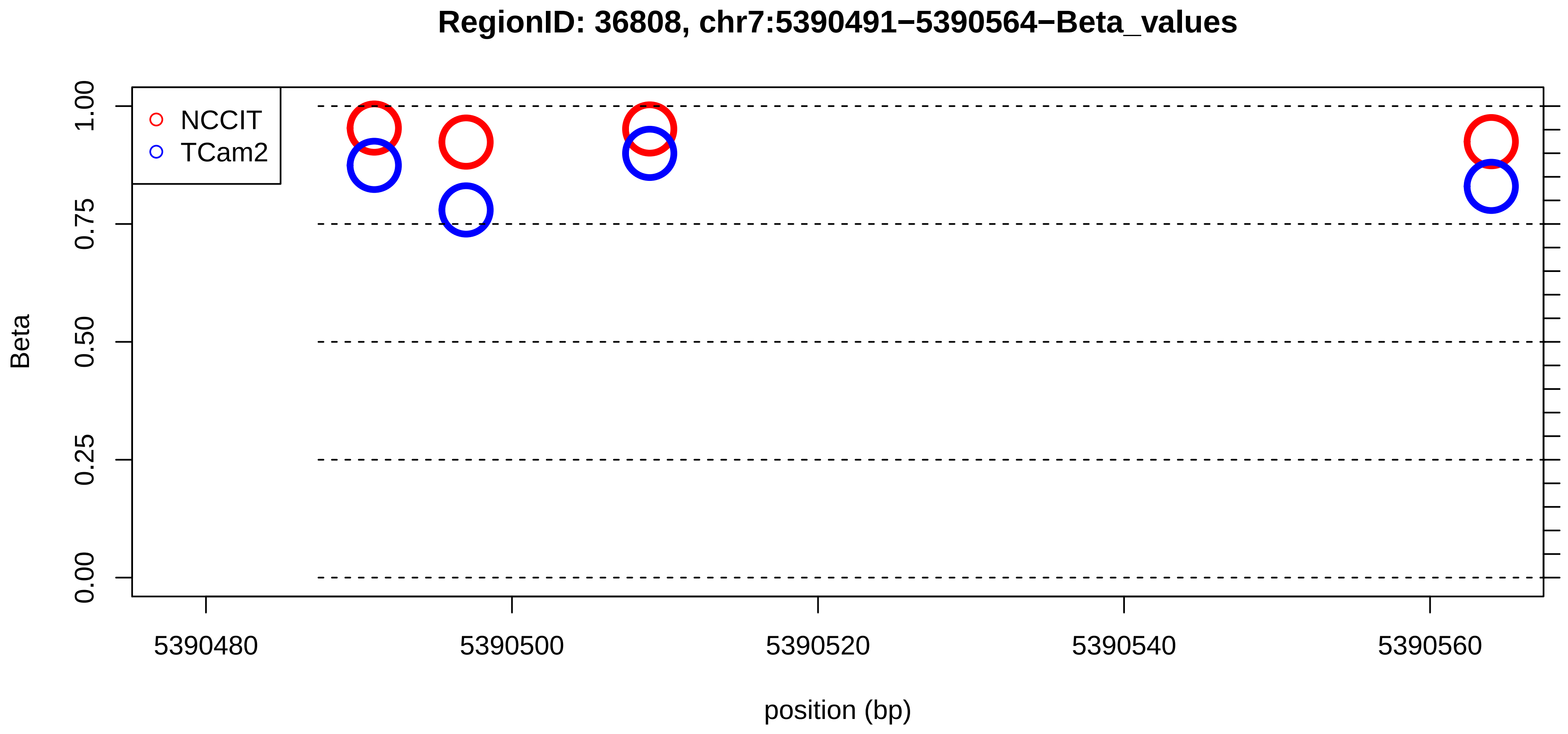
RegionID: 36808, chr7:5390491−5390564−Beta_values
NCCIT
TCam2
Beta
0.00
0.25
0.50
0.75
1.00
5390480
5390500
5390520
5390540
5390560
position (bp)
